# Composite Microbial Solid-State Fermentation Enhances the Fermentation Quality, Nutritional Value, and Safety of Cottonseed Hulls: Insights Based on Physicochemical Detection and Untargeted Metabolomics

**DOI:** 10.3390/microorganisms14071456

**Published:** 2026-07-02

**Authors:** Honghai Yang, Xiaoyan Zhou, Yuwei Ying, Yan Liu, Hanzuohere Yishake, Hongman Li, Caidie Wang

**Affiliations:** Research Center for Biofeed and Animal Gut Health, Xinjiang Herbivore Nutrition Laboratory for Meat & Milk, College of Animal Sciences, Xinjiang Agricultural University, Urumqi 830052, China; 320242693@stu.xjau.edu.cn (H.Y.); nice2cool@163.com (X.Z.); 15389911541@163.com (Y.Y.); l2077845189@163.com (Y.L.); 18099337170@163.com (H.Y.); lihong_m@163.com (H.L.)

**Keywords:** Cottonseed hulls, mixed-culture, *Aspergillus niger*, *Aspergillus oryzae*, *Lactobacillus plantarum*, gossypol detoxification, fiber degradation, feed detoxification, microbial metabolism

## Abstract

Cottonseed hulls (CSH) are by-products of cotton processing, but their use in livestock feed is constrained by lignocellulose and free gossypol. In this study, solid-state fermentation of cottonseed hulls was optimized using *Aspergillus niger*, *Aspergillus oryzae*, and *Lactobacillus plantarum* through an orthogonal experimental design. Fermentation quality, nutrient composition, safety indicators, microstructure, and metabolic profiles were subsequently evaluated under the optimized conditions. The results showed that composite microbial fermentation significantly reduced pH and decreased NH_3_-N/TN by 42.35%, while increasing lactic acid content by 20.01 g/kg. Meanwhile, butyric acid was not detected. Compared with the non-inoculated control, neutral detergent fiber and acid detergent fiber were further degraded by 5.78% and 7.37%, respectively. In addition, free gossypol was reduced by 79.79% compared with untreated cottonseed hulls, and aflatoxin B1 content decreased by 60.11% compared with the non-inoculated control. Untargeted metabolomics revealed increased abundances of amino acids and bioactive small peptides, including L-arginine, Ile-Lys, Glu-Met, and L-isoleucyl-L-arginine. Taken together, these findings indicate that composite microbial fermentation may serve as an effective strategy for the detoxification and nutritional improvement of cottonseed hulls, providing a theoretical basis for the application of fermented cottonseed hulls as a feed resource.

## 1. Introduction

Developing sustainable microbial fermentation strategies for the valorization of agro-industrial by-products is important for improving feed resource utilization and reducing environmental pressure. Cotton is a key global cash crop supporting the textile, food, and animal feed industries. Recent reports estimate that the global cotton cultivation area in the 2025 season is approximately 30 million hectares, while about 50 million tons of cotton residue waste are generated worldwide [1,2]. Cotton production and processing generate large quantities of cotton-derived residues and by-products, including cotton stalks, cottonseed meal, and cottonseed hulls (CSH). As one of the major by-products of cottonseed processing, CSH are produced in considerable quantities, particularly in major cotton-producing countries such as China, India, the United States, and Brazil. CSH are typical lignocellulosic by-products characterized by high levels of neutral detergent fiber, acid detergent fiber, and lignin, which contribute to their low digestibility and limited feeding value [3]. Their rigid and recalcitrant fiber structure impedes digestibility, thereby limiting their use in monogastric animals and certain ruminants. Additionally, CSH contain harmful antinutritional components, such as free gossypol (FG) and oxalic acid, which, when consumed in excess, can impair hepatic and renal functions and stunt animal growth [4,5,6], significantly restricting the feeding potential of CSH. Therefore, reducing fiber recalcitrance and antinutritional factors is essential for improving the feeding value and safe utilization of CSH.

In recent years, microbial fermentation has been increasingly applied to improve the nutritional value and safety of cottonseed-derived feed resources. Anaerobic solid-state fermentation with *Bacillus subtilis* has been reported to degrade free gossypol and improve the nutritional quality of cottonseed meal [7]. Similarly, solid-state fermentation with *Rhodotorula mucilaginosa* TG529 reduced free gossypol and improved the contents of crude protein, acid-soluble protein, and amino acids in cottonseed meal [8]. Microbial fermentation of cottonseed kernel has also been shown to reduce free gossypol, improve nutrient composition, and produce functional peptides and flavor-related metabolites [9]. More recently, anaerobic solid-state fermentation of CSH with *Candida utilis* CU-3 was reported to improve CSH quality by enhancing free gossypol degradation and crude protein content, reducing crude fiber content, and reshaping the fungal community and metabolite profile [10]. These findings suggest that microbial fermentation can simultaneously detoxify cotton-derived by-products, improve nutrient availability, and reshape their metabolic profiles. However, although recent studies have demonstrated the potential of microbial fermentation for cotton-derived by-products, available research on intact CSH remains limited, particularly regarding multi-strain co-fermentation systems and their integrated effects on fermentation quality, safety indicators, and metabolite remodeling.

Composite microbial fermentation is a promising strategy for enhancing the feeding quality of CSH because different microorganisms may act synergistically in lignocellulose degradation, gossypol detoxification, acidification, and nutrient transformation. *Aspergillus niger* (AN) secretes substantial quantities of cellulases and hemicellulases, effectively disrupting the rigid plant cell walls, while certain strains have been reported to utilize gossypol as the sole carbon source, exhibiting potent gossypol degradation ability [11]. Recent research on cotton stalk fermentation showed that *Aspergillus niger*-based co-fermentation significantly degraded cellulose and hemicellulose and improved the safety and nutritional value of cotton stalk feed [12]. *Aspergillus oryzae* (AO) synthesizes a variety of hydrolases, including proteases and carbohydrases, and has been demonstrated to efficiently degrade gossypol while enriching functional amino acids during solid-state fermentation of cottonseed by-products [13,14,15]. *Lactobacillus plantarum* (LP), a commonly used industrial probiotic, lowers the environmental pH rapidly through lactic acid secretion, inhibiting undesirable microbes and stabilizing the fermentation microenvironment. In addition, microbial-enzyme synergistic solid-state fermentation involving lactic acid bacteria has been shown to improve the nutritional composition and metabolite profile of cotton stalk feed [16]. Compared to single-strain fermentation, mixed microbial fermentation offers superior degradation and detoxification efficiency through the synergistic action of complementary enzyme systems and interactive metabolic regulation [17,18]. In the combined fermentation system, *Aspergillus* strains may primarily contribute to the breakdown of lignocellulose, while lactic acid bacteria regulate the fermentation conditions, significantly enhancing nutrient release and transformation [19]. Therefore, co-fermentation technology has emerged as a sustainable approach for the high-value utilization of cottonseed by-products.

Although recent studies have demonstrated the potential of microbial fermentation in cottonseed meal, cottonseed kernel, and cotton stalk, comparatively less attention has been paid to intact CSH. In particular, limited information is available on how triple-strain co-fermentation with AN, AO, and LP simultaneously affects the fermentation quality, fiber structure, gossypol detoxification, mycotoxin safety, and metabolite remodeling of CSH. To address these gaps, the present study employed a mixed fermentation system consisting of AN, AO, and LP to optimize fermentation conditions and evaluate the effects of this system on the fermentation quality, nutrient composition, safety indicators, microstructure, and metabolite profiles of CSH. In addition, untargeted metabolomics was used to identify differential metabolites resulting from composite microbial fermentation. The objective of this study was to explore the metabolic changes associated with multi-strain fermentation and to provide both theoretical and technical insights for the efficient use of fermented CSH as a feed resource in animal production. This approach may provide a sustainable strategy for converting cottonseed-processing by-products into safer and nutritionally improved feed resources.

## 2. Materials and Methods

### 2.1. Materials

CSH were purchased from Xinjiang Taikun Plant Protein Co., Ltd. (Changji, China). Bran was sourced from Xinjiang Tianshan Premixed Feed Plant (Urumqi, China). The fermentation strains, including *Aspergillus niger* (AN, 1 × 10^10^ CFU/g) and *Aspergillus oryzae* (AO, 1 × 10^10^ CFU/g), were obtained from Shandong Hezhong Kangyuan Biotechnology Co., Ltd. (Zibo, China). *Lactobacillus plantarum* (LP, 1 × 10^10^ CFU/g) was sourced from Weifang Yihao Biotechnology Co., Ltd. (Weifang, China).

### 2.2. Experimental Design

#### 2.2.1. Solid-State Fermentation of CSH

CSH and bran were weighed and thoroughly mixed. The solid-state fermentation procedure was adapted from previously reported methods for microbial fermentation of cottonseed hull-based feed and cottonseed-derived by-products, with appropriate modifications according to the characteristics of CSH and the orthogonal experimental design [16]. The specific addition amount of bran varied according to the predefined levels of the orthogonal experimental design (detailed in Section 2.2.2). Bacterial and spore suspensions were prepared by dispersing specific amounts of LP, AN, and AO into deionized water based on the designated inoculation rates for each experimental group. The inoculation rate was defined as the mass ratio of the combined microbial agents to the air-dried weight of the substrate. The suspensions were then uniformly sprayed onto the substrate mixture, adjusting the initial moisture content to the specific levels dictated by the experimental design. Subsequently, the mixture was transferred into 5 L respiration bags, sealed, and incubated at ambient temperature (28–35 °C) for 15 days. Following fermentation, 10 g of fermented cottonseed hulls was homogenized with 90 mL of sterile water to prepare an aqueous extract. The extract was used immediately for pH determination, and an aliquot of the filtrate was transferred into 2 mL cryogenic vials and stored at −20 °C for subsequent ammonia nitrogen and organic acid analyses. In addition, approximately 200 g of fermented cottonseed hulls was collected and dried at 105 °C for 30 min to inactivate enzymes and microorganisms, followed by drying at 65 °C to a constant weight. The dried samples were ground and passed through a 40-mesh sieve prior to further analyses.

#### 2.2.2. Orthogonal Experimental Design

An L_9_(3^4^) orthogonal experimental design was employed to optimize the solid-state fermentation parameters (Table 1). The three designated factors evaluated were initial moisture content, bran addition amount, and inoculation rate. The fourth column was left empty as an error column to estimate random errors and assess experimental reliability. Each treatment was performed in hexaplicate (*n* = 6) for a 15-day fermentation period. For the orthogonal analysis, the mean value of six replicates for each treatment was used to calculate the composite index and conduct range analysis. Fermentation performance was evaluated using six observation indicators: degradation rates of FG, acid detergent fiber (ADF) and neutral detergent fiber (NDF), pH, the ratio of ammoniacal nitrogen to total nitrogen, and the Flieg’s score. The degradation rates of FG, NDF, and ADF were calculated according to the following equations:

(1) $$FG\;degradation\;rate\left(\%\right)=[({FG}_{0}-\;{FG}_{t})\;/\;{FG}_{0}]\times 100$$

(2) $$NDF\;degradation\;rate\;(\%)=[({NDF}_{0}-{NDF}_{t})\;/\;{NDF}_{0}]\times 100$$

(3) $$ADF\;degradation\;rate\;(\%)=[({ADF}_{0}-{ADF}_{t})/\;{ADF}_{0}]\times 100$$

where *FG*_0_, *NDF*_0_, and *ADF*_0_ represent the initial contents of free gossypol, neutral detergent fiber, and acid detergent fiber before fermentation, respectively; *FG*_t_, *NDF*_t_, and *ADF*_t_ represent their corresponding contents after fermentation.

To resolve potential incompatibilities among these multiple indicators and simplify data interpretation, a composite index calculation method (*F*) was adopted [20]. The raw data for each indicator were first standardized. For indicators where higher values are desirable (degradation rates of FG, ADF, NDF, and Flieg’s score), data were standardized using Equation (4):

(4) $$I_{i}=\frac{X_{i}-X_{min}}{X_{max}-X_{min}}$$

For indicators where lower values are optimal (ammoniacal nitrogen to total nitrogen ratio and pH), Equation (5) was applied:

(5) $$I_{i}=\frac{X_{max}-X_{i}}{X_{max}-X_{min}}$$

where *I_i_* represents the standardized value, *X_i_* is the mean of the six replicates, and *X_max_* and *X_min_* are the maximum and minimum values of that parameter, respectively. Finally, a composite index *F* was calculated according to Equation (6):

(6) $$F=\sum \left(W_{i}\times I_{i}\right)\times 100$$

where *W_i_* is the weighting coefficient (Σ*_Wi_* = 1, Table 2), assigned to reflect the relative importance of parameters such as gossypol reduction and digestibility. A higher *F* denotes superior fermentation performance.

#### 2.2.3. Validation of the Optimal Fermentation Process

To validate the optimized parameters, three experimental configurations were established: an untreated group (UG; unfermented CSH), a control group (CG; CSH supplemented with 20% bran and adjusted to 55% moisture without microbial inoculation), and an experimental group (EG; CSH supplemented with 20% bran, adjusted to 55% moisture, and inoculated with the microbial mixture at 0.1%). Fermentation was conducted for 15 days under the optimal conditions identified in Section 2.2.2. Subsequently, the fermented products were comprehensively evaluated for quality, microstructure, nutrient profile, and toxin degradation.

### 2.3. Determination of Fermentation Quality

Sensory evaluation was conducted according to the German Agricultural Society (DLG) silage standards (color, odor, and texture; max 20 points). pH was measured using a Mettler FE28 pH meter (Mettler-Toledo, Columbus, OH, USA). Ammonia nitrogen concentration was determined using a Pentra 400 analyzer (Horiba Ltd., Kyoto, Japan) following the method of Rhine et al. [21]. Referring to the Förster (Flieg’s score) scoring method for silage feed by Guo et al. [22], the organic acid score was calculated based on the proportions of lactic acid, acetic acid, and butyric acid in the total acid content. The contents of acetic acid, propionic acid, butyric acid, and lactic acid were determined using high-performance liquid chromatography (HPLC; Shimadzu LC-40, Shimadzu Corporation, Kyoto, Japan). The HPLC method was adapted from a Thermo Fisher Scientific AppsLab application for monocarboxylic acid analysis using an Acclaim Mixed-Mode WAX-1 column, with modifications according to the characteristics of CSH fermentation extracts [23]. Briefly, the sample extracts stored at −20 °C were thawed at 4 °C. After thawing, 1.5 mL of each extract was transferred into a 2 mL centrifuge tube and centrifuged at 12,000 rpm for 10 min at 4 °C. The supernatant was filtered through a 0.22 μm membrane filter and transferred into a 1.5 mL brown glass vial for HPLC analysis. The chromatographic conditions were as follows: Thermo Fisher Acclaim WAX-1 column (4.6 × 250 mm); mobile phase, acetonitrile:25 mM potassium dihydrogen phosphate buffer (pH adjusted to 3.93 with phosphoric acid) = 25:75 (*v*/*v*); column temperature, 30 °C; flow rate, 0.8 mL/min; UV detection wavelength, 210 nm; injection volume, 10 μL; isocratic elution; and run time, 20 min.

### 2.4. Determination of the Microstructure of CSH

Samples of CSH from the raw material, control, and experimental groups were prepared for scanning electron microscopy (SEM) observation according to previously reported methods for fermented cotton-derived lignocellulosic materials, with minor modifications [24]. The samples were sputter-coated with gold to provide conductivity. The microstructures were then observed using a scanning electron microscope (SU8010; Hitachi High-Tech Corp., Tokyo, Japan) at a magnification of 2500× under vacuum conditions.

### 2.5. Routine Nutritional Analysis

Ash, crude fat, moisture, and crude protein contents were determined according to the AOAC procedures [25]. Neutral detergent fiber (NDF) and acid detergent fiber (ADF) were analyzed according to Van Soest et al. [26]. Calcium and phosphorus contents were determined using the method described by Hambleton [27].

### 2.6. Determination of FG and Mycotoxin Content

FG content was determined by HPLC (Shimadzu LC-40, Kyoto, Japan) using an analytical column: C18 column (250 mm × 4.6 mm, 5 μm). The mobile phase consisted of acetonitrile and 0.2% phosphoric acid (85:15, *v*/*v*) at a flow rate of 1 mL/min, with detection at 235 nm [28]. Aflatoxin B1 (AFB1) and ochratoxin A (OTA) were analyzed using an FD-800 rapid fluorescent quantitative mycotoxin analyzer (Shanghai Feice Bio-Tech Co., Ltd., Shanghai, China) according to the manufacturer’s instructions. The limits of detection were 1 μg/kg for AFB1 and 10 μg/kg for OTA, and results below the detection limit were recorded as not detected.

### 2.7. Determination of Metabolites

For each group, six biological replicates were analyzed. Untargeted metabolomic analysis of cottonseed hull samples from the control and experimental groups was performed by Novogene Co., Ltd. (Beijing, China) using its standard UHPLC-MS/MS-based metabolomics workflow, with minor modifications according to a previously reported tissue metabolomics protocol [29]. Briefly, 100 mg of each sample was ground in liquid nitrogen and extracted with pre-cooled 80% methanol. The mixture was vortexed, incubated on ice for 5 min, and centrifuged at 15,000× *g* for 20 min at 4 °C. A portion of the supernatant was diluted with LC-MS-grade water to obtain a final methanol concentration of 53%, followed by centrifugation at 15,000× *g* for 20 min at 4 °C. The final supernatant was collected for LC-MS/MS analysis. Quality control samples were prepared by pooling equal aliquots from all samples.

UHPLC-MS/MS analysis was performed using a Vanquish UHPLC system coupled with an Orbitrap high-resolution mass spectrometer. Chromatographic separation was carried out on a Hypersil Gold column (100 mm × 2.1 mm, 1.9 μm) at a flow rate of 0.2 mL/min. The mobile phases consisted of 0.1% formic acid in water as solvent A and methanol as solvent B. A 12 min linear gradient was used as follows: 0–1.5 min, 2% B, 1.5–3 min, 2–85% B, 3–10 min, 85–100% B, 10–10.1 min, 100–2% B, and 10–12 min, 2% B. The mass spectrometer was operated in both positive and negative ion modes with a spray voltage of 3.5 kV, capillary temperature of 320 °C, sheath gas and auxiliary gas flow rates of 35 and 10 arbitrary units, S-lens RF level of 60, and auxiliary gas heater temperature of 350 °C.

Raw data generated from UHPLC-MS/MS were processed using XCMS for peak detection, alignment, and quantification [30]. Metabolites were identified by matching accurate mass, adduct ions, and MS/MS spectra against NovoMetDB, a self-built high-quality secondary spectrum database with a mass tolerance of 10 ppm. Metabolites with more than 50% missing values were removed, and the remaining missing values were imputed using the K-nearest neighbor method [31]. Compounds with a coefficient of variation greater than 30% in quality control (QC) samples were excluded.

Metabolites were annotated using the KEGG, HMDB, and LIPIDMaps databases [32,33,34]. Principal component analysis (PCA) and partial least squares discriminant analysis (PLS-DA) were performed using metaX software (version 2.0.0) [35]. Differential metabolites were screened based on variable importance in projection (VIP) > 1.0, fold change > 1.5 or <0.667, and *p* < 0.05. KEGG pathway enrichment analysis was performed to identify significantly enriched pathways. Spearman correlation analysis between differential metabolites and physicochemical parameters was conducted using OriginPro 2024, and *p* < 0.05 was considered statistically significant.

### 2.8. Statistical Analysis

Statistical analyses were conducted using two specialized software packages. For the orthogonal experimental design, range analysis and analysis of variance (ANOVA) were performed using the General Linear Model (GLM) univariate procedure in IBM SPSS Statistics software (version 27.0, IBM Corp., Armonk, NY, USA). For the validation and group comparison experiments, all statistical analyses were carried out using SAS software (version 9.4, SAS Institute Inc., Cary, NC, USA). Specifically, differences between two independent groups were analyzed using Student’s *t*-test. For comparisons involving three or more groups, data were subjected to one-way ANOVA, followed by Duncan’s multiple range test for post hoc mean comparisons to identify significant differences between specific treatments. All experimental results were expressed as mean ± standard deviation (SD), and the threshold for statistical significance was set at *p* < 0.05.

## 3. Results

### 3.1. Results of the Orthogonal Experiment

According to the range analysis presented in Table 3, the effects of the tested factors on the composite index followed the order: bran addition ratio (B) > inoculum size (C) > moisture content (A) > blank column (D). The relatively small range value observed for the blank column (D) indicated acceptable random error and good consistency of the experimental data. Considering the technical requirements for CSH fermentation with the composite microbial inoculant, the optimal parameter combination was determined to be B3C2A1. Specifically, the ideal conditions were established as a 20% bran addition, 55% moisture content, and a 0.1% inoculum level. The ANOVA results presented in Table 4 further showed that bran addition ratio, inoculum size, and moisture content had significant effects on the composite index (*p* < 0.05).

### 3.2. Effects of Composite Microbial Fermentation on the Fermentation Quality of CSH

As shown in Table 5, inoculation with the composite microbial markedly improved the sensory quality of CSH compared with the control group (CG). The experimental group (EG) exhibited superior odor, color, and texture. Consequently, the EG achieved a “good” sensory rating and a Flieg’s score of 98.17, classifying its fermentation quality as “excellent”.

As shown in Table 6, the pH, NH_3_-N/TN ratio, acetic acid content, and NH_3_-N content in the EG were significantly lower than those in the CG (*p* < 0.05). In contrast, lactic acid and total acid contents were significantly increased in the EG (*p* < 0.05), with the lactic acid content reaching 2.51 times that of the CG. Notably, butyric acid was not detected in the EG, indicating improved fermentation quality. Relative to the CG, the EG was characterized by a 42.35% reduction in NH_3_-N/TN, together with a 20.01 g/kg elevation in lactic acid content and a 57.38% enhancement in total acid accumulation.

### 3.3. Effects of Composite Microbial Fermentation on the Microstructure of CSH

**Figure 1:**
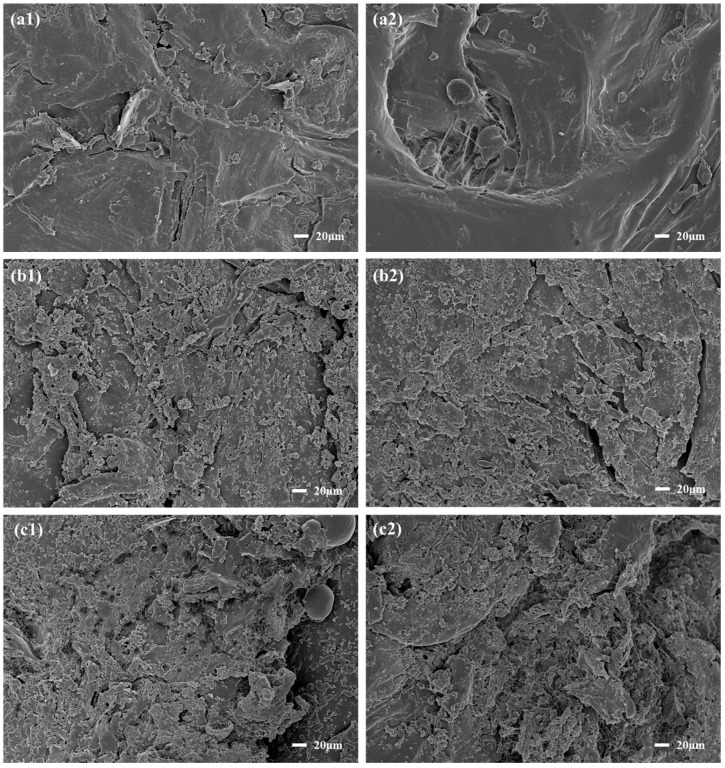
Scanning electron microscope images of cotton seed hulls subjected to different treatments. (**a1**,**a2**) Cottonseed hulls from the untreated group (UG). (**b1**,**b2**) cottonseed hulls from the control group (CG). (**c1**,**c2**) cottonseed hulls from the experimental group (EG). The images were obtained at a voltage of 3.0 kV and a magnification of 2500× under an electron microscope.

Under 2500× scanning electron microscopy, the surface structure of unfermented CSH appeared dense, with large, closely packed blocks and no obvious pores or loose areas (Figure 1(a1,a2)). Compared with the CG, the EG exhibited more pores. Following solid-state fermentation with the LP, AN, and AO mixture (Figure 1(c1,c2)), microbial-like structures were observed on the surface of CSH, and the microstructure transformed into a loose, reticulated morphology with distinct pores.

### 3.4. Effects of Composite Microbial Fermentation on the Nutrient Composition of CSH

As shown in Table 7, composite microbial fermentation altered the nutrient composition of CSH. Following the 15-day fermentation period, no significant differences were observed between the EG and CG regarding crude protein (CP), ether extract (EE), and calcium contents (*p* > 0.05). However, the EG displayed significantly lower neutral detergent fiber (NDF) and acid detergent fiber (ADF) contents than the CG (*p* < 0.05). Compared with the CG, NDF and ADF were further reduced by 5.78% and 7.37%, respectively, indicating an improvement in the fiber profile of fermented CSH.

When evaluated against the untreated group (UG), the EG displayed a significant elevation in CP content (*p* < 0.05), alongside a sharp decline in both NDF and ADF levels (*p* < 0.05). These findings demonstrate that composite microbial fermentation effectively degraded the complex structural fibers within CSH, indicating an improvement in the potential feeding value of CSH.

### 3.5. Effects of Composite Microbial Fermentation on Free Gossypol and Mycotoxin Contents in CSH

As shown in Table 8, composite microbial fermentation significantly improved the safety-related indicators of CSH. The FG content in the EG was significantly lower than that in both the UG and CG (*p* < 0.05), and the FG content was reduced by 79.79% compared with the UG. In addition, the aflatoxin B1 content in the EG was significantly lower than that in the CG (*p* < 0.05), corresponding to a 60.11% decrease, while ochratoxin A was below the detection limit in all groups. Compared with the CG, the EG further reduced FG content by 41.03%, indicating that inoculation with the composite microbial consortium provided additional detoxification relative to the non-inoculated fermentation treatment.

### 3.6. Effects of Composite Microbial Fermentation on Metabolic Changes in CSH

#### 3.6.1. QC Sample Analysis

**Figure 2:**
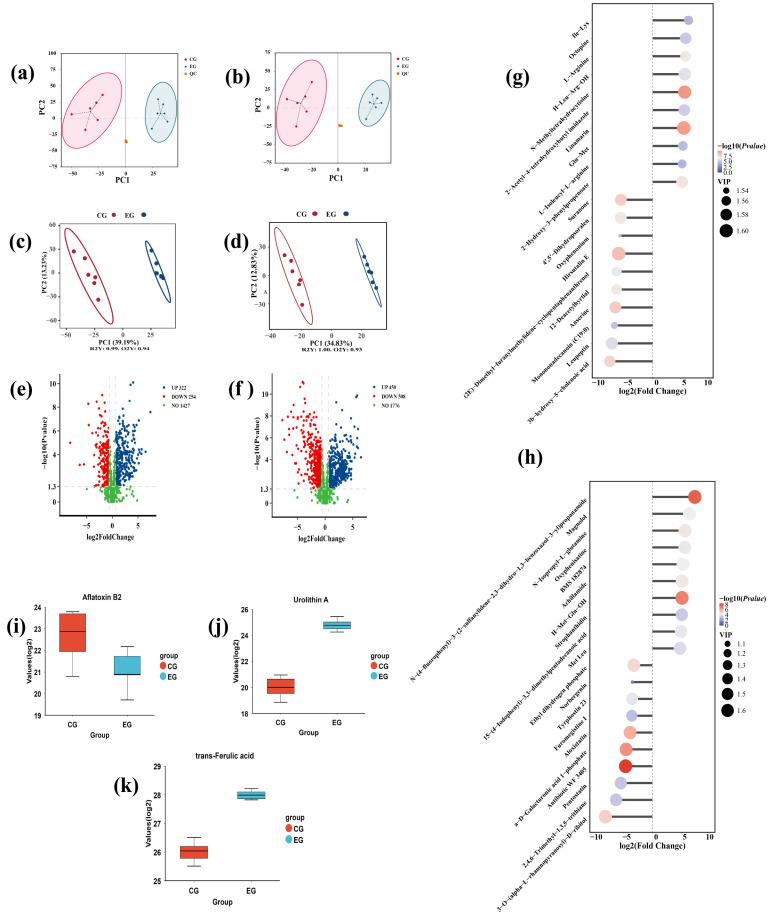
Analysis of differential metabolites (*n* = 6). (**a**,**b**) Principal Component Analysis (PCA) score plots in positive and negative ion modes. The QC samples consist of mixtures of equal volumes of all samples; the first three QC samples, introduced prior to sample injection, are used to monitor the instrument’s condition and equilibrate the chromatography-mass spectrometry system, while the QC samples inserted during the sample analysis are used to evaluate system stability throughout the experiment and to perform data quality control analysis. (**c**,**d**) Partial Least Squares Discriminant Analysis (PLS-DA) score plots in positive and negative ion modes. (**e**,**f**) Volcano plots of differential metabolites in positive and negative ion modes. (**g**,**h**) Fold change and VIP score plots of differential metabolites in positive and negative ion modes. (**i**) Relative abundance of Aflatoxin B2; (**j**) Relative abundance of Urolithin A. (**k**) Relative abundance of trans-Ferulic acid.

As shown in Figure 2a,b, samples from the CG and EG exhibited distinct separation in both positive and negative ion modes, whereas the QC samples remained tightly clustered. This indicates that the analytical process was stable and the data generated were of high quality.

#### 3.6.2. Differential Metabolite Screening Results

To identify metabolic divergence between the CG and EG, a partial least squares discriminant analysis (PLS-DA) model was constructed. As illustrated in the score plots (Figure 2c,d), the CG and EG samples were clearly separated in the positive ion mode, indicating clear group discrimination and significant metabolic shifts. The R^2^_Y_ and Q^2^_Y_ values were 0.99 and 0.94, respectively. In the negative ion mode, clear segregation was also observed, with R^2^_Y_ and Q^2^_Y_ values of 1.00 and 0.93, indicating strong discriminative performance in this dataset. Differential metabolites (DEMs) were screened based on VIP > 1.0, FC > 1.5 or <0.667, and *p* < 0.05 (Figure 2e,f). Compared with the CG, the EG showed 958 DEMs in the positive ion mode, including 450 increased and 508 decreased metabolites, and 576 DEMs in the negative ion mode, including 322 increased and 254 decreased metabolites. In the positive ion mode, the EG exhibited higher abundances of Ile-Lys, Octopine, L-Arginine, and Glu-Met, whereas 3β-hydroxy-5-cholenoic acid, Leupeptin, and 12-Deacetylhyrtial showed lower abundances (Figure 2g). In the negative ion mode, Magnolol, N-Isopropyl-L-glutamine, and H-Met-Glu-OH were enriched in the EG, whereas 3-O-(α-L-rhamnopyranosyl)-D-ribitol, Pentostatin, and Norbergenin were decreased (Figure 2h). In addition, the relative abundance of putatively annotated aflatoxin B2 was decreased in the EG, which was in line with the improved mycotoxin-related safety indicators observed after fermentation (Figure 2i). Additionally, urolithin A and trans-ferulic acid showed significantly higher abundances in the EG (Figure 2j,k).

#### 3.6.3. KEGG Pathway Enrichment Analysis and Correlation Analysis Between Differential Metabolites and Physicochemical Properties

**Figure 3:**
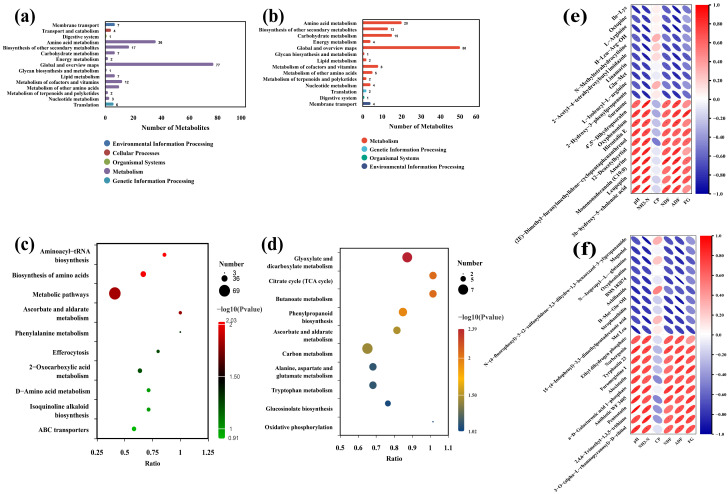
KEGG enrichment analysis and correlation analysis plot of differential metabolites and physicochemical properties (*n* = 6). (**a**) KEGG pathway classification bar chart of differential metabolites in positive ion mode. (**b**) KEGG pathway classification bar chart of differential metabolites in negative ion mode. (**c**) Top 10 enriched KEGG pathways of differential metabolites in positive ion mode. (**d**) Top 10 enriched KEGG pathways of differential metabolites in negative ion mode. (**e**) Spearman correlation heatmap between the top ranked significant differential metabolites in positive ion mode and physicochemical parameters. (**f**) Spearman correlation heatmap between the top ranked significant differential metabolites in negative ion mode and physicochemical parameters, * *p* ≤ 0.05, ** *p* ≤ 0.01, *** *p* ≤ 0.001.

Figure 3a presents the KEGG functional annotation and classification of DEMs in the positive ion mode. The largest number of DEMs (77) was assigned to “Global and overview maps,” followed by amino acid metabolism. A similar trend was observed in the negative ion mode (Figure 3b), where “Global and overview maps” predominated with 50 DEMs, followed by amino acid metabolism and carbohydrate metabolism. Figure 3c,d display the top 10 significantly enriched metabolic pathways. In the positive ion mode, five pathways were significantly enriched, including aminoacyl-tRNA biosynthesis, biosynthesis of amino acids, and metabolic pathways. In the negative ion mode, six pathways were significantly enriched: glyoxylate and dicarboxylate metabolism, the citrate cycle (TCA cycle), butanoate metabolism, phenylpropanoid biosynthesis, ascorbate and aldarate metabolism, and carbon metabolism. Correlation analysis showed that after cottonseed hulls were fermented by AN, AO, and LP (Figure 3e,f), the differential metabolites were closely associated with physicochemical properties. In both positive and negative ion modes, selected amino acids, peptides, and their derivatives, such as Ile-Lys, L-Arginine, Glu-Met, H-Met-Glu-OH, and Magnolol, were positively correlated with CP, but negatively correlated with NDF, ADF, NH_3_-N, and FG.

## 4. Discussion

### 4.1. The Effect of Composite Microbial on the Fermentation Quality of CSH

In this study, a 1:1:1 mixture of AN, AO, and LP was used for the co-fermentation of CSH, which significantly improved the sensory quality of the fermented CSH, making them superior to the control group in terms of odor, color and texture. After 15 days of fermentation, relative to the control group, inoculation significantly decreased pH and reduced the NH_3_-N/TN ratio by 42.35%, while increasing lactic acid content by 20.01 g/kg. Collectively, these changes indicate that the consortium promoted stronger acidification and suppressed nitrogen degradation. The ammoniacal nitrogen content and the ratio of ammoniacal nitrogen to total nitrogen in the experimental group’s CSH were significantly lower than those in the CG, while butyric acid was not detected. This indicates that the composite microbial inoculant effectively promotes lactic acid production, rapidly lowers the pH of the fermentation environment, and inhibits the growth and reproduction of putrefactive and ammonia-producing bacteria. This also explains the good stability of the experimental group’s fermentation process in this study. Overall, the fermentation process in the EG was easier to control. This improvement was consistent with the known role of LP in promoting lactic acid accumulation, lowering pH, and suppressing undesirable microorganisms during plant-material fermentation [36,37]. AN and AO may have further supported this process by increasing the availability of fermentable substrates through the hydrolysis of structural carbohydrates and macromolecular nutrients. In addition, co-cultivation of AN and AO has been reported to enhance the production of lignocellulose-degrading enzymes, including β-glucosidase, cellobiohydrolase, β-xylosidase, and laccase [38]. Therefore, the improvement in fermentation quality observed in the experimental group was likely associated with the combined effects of fungal substrate hydrolysis and LP-mediated acidification, rather than the independent action of a single strain. The above results are consistent with those reported by Li et al. [12] regarding the fermentation of cotton stalks using *Aspergillus niger*, *Bacillus licheniformis* and *Lactobacillus casei*. In this study, the acetic acid content in the experimental group’s CSH was significantly lower than that in the control group. Acetic acid is a common product of microbial fermentation; some lactic acid bacteria produce acetic acid and carbon dioxide in addition to lactic acid when metabolising carbohydrates. The high acetic acid content in the control group’s CSH may be attributed to the presence of acetic acid-producing microorganisms in the raw material, which were able to grow and metabolize in the absence of competition. By contrast, the experimental group was characterized by higher lactic acid and total acid accumulation, together with a lower acetic acid level, indicating a shift toward lactic acid-dominated fermentation, in the EG, AN and AO provided more fermentable sugars through substrate hydrolysis, while *Lactobacillus plantarum*, as the dominant strain, produced lactic acid as its primary metabolite. This rapid acidification process, dominated by *Lactobacillus plantarum*, may have inhibited the growth of the original acetic acid-producing bacteria and the production of acetic acid in the system.

### 4.2. The Effect of Composite Microbial Fermentation on the Nutrient Content of CSH

AN, AO, and LP possess the ability to increase cellular protein content, break down proteins into smaller molecules, and degrade fiber. Research has found that AN secretes a wide variety of proteases, including aspartic proteases, serine proteases and metalloproteases [39,40]. These enzymes act synergistically to achieve the gradual degradation of proteins. During fermentation, proteases secreted by microorganisms hydrolyze macromolecular proteins into small peptides and free amino acids, thereby improving the amino acid profile of the feed and enhancing protein digestibility and utilization [41]. In solid-state fermentation, AN significantly reduces the crude fiber, NDF and ADF content in various oilseed meals [42], effectively improving their nutritional composition and increasing in vitro digestibility [43]. Wang et al. [44] found during the preparation of paper mulberry silage that the addition of LP and bran could increase crude protein content, reduce pH and ammoniacal nitrogen levels, and improve fermentation quality. In this study, there was no significant difference in crude protein content between the experimental and control groups’ CSH; however, the crude protein content in both the experimental and control groups’ CSH was higher than that in the untreated group. This is consistent with the findings of Li et al. [45], where the protein content of CSH increased relatively as fermentation time was extended. This elevation is likely a combined result of microbial protein synthesis and the “concentration effect” triggered by the consumption of soluble carbohydrates during solid-state fermentation, which increases the relative proportion of nitrogen [46]. During fermentation, microorganisms secrete proteases to hydrolyze macromolecular proteins in the substrate, producing small peptides and free amino acids. These products are then absorbed by the microorganisms as a nitrogen source for the synthesis of their own cellular proteins, leading to an increase in crude protein content [41]. Although the DM percentage remained stable across groups (indicating consistent moisture levels), the relative proportions of ash and phosphorus increased. This may be because the microbial degradation of organic components reduced the total dry matter mass, thereby concentrating the non-volatile inorganic minerals within the remaining substrate. In the present study, microbial inoculation resulted in 5.78% and 7.37% reductions in NDF and ADF, respectively, relative to the control group. These reductions suggest that the inoculated consortium enhanced the accessibility and utilization of lignocellulosic components in CSH, thereby improving its potential feeding value. Moreover, SEM observations (2500×) revealed a loose, porous, and reticulated microstructure. Together, these findings confirm the effective disruption of the lignocellulosic barrier by the microbial consortium, consistent with the morphological changes reported in fermented cotton straw by Hu et al. [24]. These results indicate that the improvement in nutritional value was not limited to changes in conventional nutrient composition, but was also related to structural modification of CSH. Similar decreases in cellulose and hemicellulose have been reported during *Aspergillus niger*-based fermentation of cotton stalks, suggesting that fungal fermentation can contribute to the degradation of cotton-derived lignocellulosic substrates [12]. The partial disruption of the compact fiber structure may increase substrate accessibility and facilitate subsequent microbial or digestive enzyme action, although this potential effect requires further in vivo verification.

### 4.3. The Effect of Composite Microbial Fermentation on the Levels of FG and Mycotoxins in CSH

In this study, fermentation reduced the FG content in CSH, and inoculation with AN, AO, and LP further enhanced this reduction. The FG content in the control group was significantly lower than that in the untreated group, indicating that spontaneous fermentation contributed to FG reduction. Under the same bran addition and moisture conditions, microbial inoculation achieved an additional 41.02% reduction in FG relative to the control group. This comparison indicates that the inoculated microbial consortium contributed additional detoxification beyond the effects of spontaneous fermentation and substrate adjustment. Relative to the untreated group, FG content was reduced by 79.79% in the experimental group. This substantial reduction indicates that composite microbial fermentation effectively mitigated this major anti-nutritional factor. Cotton stalks, cottonseed meal, and CSH naturally contain diverse microorganisms, some of which may have the ability to degrade FG [47]. Nevertheless, fermentation without microbial inoculation may also allow undesirable microorganisms to proliferate, potentially reducing feed quality and safety. Previous studies have shown that microbial fermentation can reduce FG in cottonseed-derived feed materials [8]. AO has been reported to reduce FG in cottonseed meal [48], and Chen et al. [15] used AO to ferment crushed CSH, achieving an FG degradation rate of 78.8%. Some AN strains can secrete low-activity phenol oxidases and reduce gossypol toxicity by modifying its hydroxyl groups [49]. In addition, mixed microbial fermentation has been shown to reduce FG in cottonseed meal; for example, the combined use of *Aspergillus niger*, *Aspergillus oryzae*, and *Bacillus subtilis* reduced FG content by up to 88.36% [50]. The FG degradation rate observed in the present study was comparable to these previous reports, despite the intact and compact structure of CSH, which may limit microbial and enzymatic access to FG embedded within the substrate matrix. Moreover, free gossypol can react with amino groups to form bound gossypol, which is generally considered less readily absorbed and less toxic than free gossypol [51]. Therefore, the FG reduction observed in this study may be associated with microbial transformation, enzymatic modification, and conversion into less reactive bound forms. However, the exact degradation products were not identified and require further investigation.

In addition to FG reduction, composite microbial fermentation also affected mycotoxin-related safety indicators. In this study, the AFB1 content in the control group increased significantly compared with the raw material, suggesting that spontaneous fermentation may have favored the proliferation or metabolic activity of aflatoxin-producing microorganisms. By comparison, the experimental group exhibited a 60.11% lower AFB1 level than the control group, suggesting that inoculation with AN, AO, and LP helped limit AFB1 accumulation during fermentation. Wang et al. [52] reported that co-cultivation of AN and Pleurotus ostreatus achieved an AFB1 degradation rate of up to 93.4%. The lower AFB1 level in the experimental group may be related to acidification-mediated inhibition of toxin-producing microorganisms, microbial adsorption, or biotransformation of aflatoxins, as reported for lactic acid bacteria and other microorganisms [53]. However, this study did not directly determine AFB1 degradation products, microbial adsorption capacity, or detoxification enzymes. Therefore, the reduction in AFB1 should be interpreted as an improvement in safety-related indicators rather than direct evidence for a specific detoxification pathway.

### 4.4. The Effect of Composite Microbial Fermentation on Metabolic Profiles

In this study, multivariate statistical analyses, including PCA and PLS-DA score plots, revealed a distinct separation in the metabolic profiles between the control group and experimental group. This separation indicates that the composite microbial consortium altered the overall metabolic profile of CSH rather than only affecting several individual compounds. The identification of 958 and 576 differential metabolites in positive and negative ion modes, respectively, indicates extensive metabolic remodeling induced by composite microbial fermentation. Increased levels of amino acids and amino acid-derived peptides, such as Ile-Lys, L-arginine, L-isoleucyl-L-arginine, Glu-Met, and Met-Leu, were observed in both ion modes. This accumulation likely stems from the proteolytic activity of *Aspergillus* species, which degrade CSH proteins into accessible nitrogen sources. This improvement in protein-related metabolites may contribute to the nutritional value of fermented CSH.

In addition, physicochemical analysis demonstrated a significant reduction in FG in the fermented CSH. This reduction was accompanied by a decrease in the relative abundance of annotated aflatoxin B2, as suggested by untargeted metabolomic analysis. Although FG itself was not directly annotated in the non-targeted metabolome, its detoxification can be partially elucidated through metabolic shifts and microbial mechanisms. The significant negative correlation between macroscopic FG levels and the increased free amino acids suggests a potential chemical detoxification pathway: the reactive aldehyde groups of FG may undergo Schiff base reactions with microbially released amino groups, converting FG into bound gossypol [54]. Concurrently, the reduction in Aflatoxin B2 and FG may also be attributed to the synergistic effect of the microbial consortium, involving physical binding by the peptidoglycan network of the *Lactobacillus plantarum* cell wall [55], and potential enzymatic biotransformation by oxidoreductases secreted during fungal growth.

The biotransformation of the CSH matrix is further supported by the significant accumulation of trans-Ferulic acid. This metabolite serves as a key indicator of the enzymatic cleavage of ester bonds linking lignin and hemicellulose, likely driven by the cellulolytic and hemicellulolytic enzyme systems, such as feruloyl esterases, of AN [56]. Furthermore, the fermentation process enriched functional small-molecule metabolites such as urolithin A and magnolol, suggesting the microbially mediated bioconversion of complex polyphenolic precursors. Shen et al. [57] demonstrated that magnolol (25, 50, 100 mg/kg) alleviates inflammation and mucosal damage in DSS-induced UC mice by downregulating TNF-α, IL-1β and IL-12, upregulating PPARγ, inhibiting NF-κB, and upregulating the expression of ZO-1 and occludin, suggesting that composite fermentation may enhance the functional potential of the product [16]. As shown in the correlation heatmaps, these microbially driven metabolic shifts, specifically the accumulation of amino acids, peptides, and potentially bioactive compounds, were positively correlated with CP and negatively correlated with NDF, ADF, NH_3_-N, and FG. These correlations provide an integrated explanation linking metabolite remodeling with macroscopic improvements in fermentation quality, fiber degradation, and safety indicators. In their study on fermented cottonseed meal, Niu et al. [58] identified 104 differential metabolites using non-targeted metabolomics, with levels of beneficial metabolites such as L-methionine, L-glutamine, ornithine and citrulline increasing following fermentation. Consistent with this, our KEGG functional classification indicates that the differential metabolites are mainly enriched in carbohydrate and amino acid metabolism pathways. This reflects the metabolic interplay within the consortium: AO and AN deconstruct lignocellulose and proteins, providing fermentable sugars and amino acids. This may create a favorable substrate environment for LP and contribute to changes in energy and organic acid metabolism, as reflected by the enrichment of TCA cycle- and butanoate metabolism-related pathways [59]. Ultimately, this microbial synergy modifies the carbon and nitrogen nutritional components of CSH, providing a metabolomic basis for its application as a feed ingredient.

From a sustainability perspective, composite microbial fermentation provides a feasible route for the bioconversion of cottonseed hulls, reducing antinutritional risks while improving their potential value as unconventional feed ingredients.

### 4.5. Study Limitations

In summary, the key to designing an efficient fermentation process lies in establishing a microbial community with complementary functions, rather than simply combining strains with single functions. Future research should further quantify the distribution of each strain within the co-culture and explore a wider variety of microbial combinations to achieve effective control over the fermentation process. In this study, the metabolic process of CSH fermentation using composite microbial not only reduced the content of FG and improved fiber quality, but also increased the levels of certain bioactive compounds; future research should focus on the specific biotransformation pathways of complex metabolites and their long-term efficacy in animal production.

## 5. Conclusions

The optimal conditions for solid-state fermentation of CSH with AN, AO, and LP were determined as follows: bran addition of 20%, inoculum size of 0.1%, and moisture content of 55%. Under these conditions, pH and ammoniacal nitrogen levels were significantly reduced, lactic acid content increased, and butyric acid was not detected, indicating a marked improvement in fermentation quality. Meanwhile, the contents of NDF and ADF decreased significantly, and the levels of FG and aflatoxins were reduced, thereby enhancing feed safety and nutritional value. Untargeted metabolomics revealed significantly increased abundances of amino acids and bioactive small peptides, as well as significant enrichment of pathways such as carbohydrate metabolism, amino acid metabolism, and the TCA cycle. These findings indicate that composite microbial fermentation effectively reconfigures the carbon and nitrogen components of CSH and generates beneficial metabolites, suggesting improved potential as a feed ingredient.

## Figures and Tables

**Table 1:** Table of factor levels for an orthogonal experiment.

Level	Factor		
	Moisture Content (A, %)	Bran Ratio (B, %)	Inoculum Size (C, %)
1	55	10	0.05
2	60	15	0.10
3	65	20	0.15

**Table 2:** Weighting coefficients for each indicator.

Parameter	Weighting Factor
Free gossypol degradation rate	0.2
Neutral detergent fiber degradation rate	0.2
Acid detergent fiber degradation rate	0.2
Ammonia nitrogen/total nitrogen	0.1
pH	0.1
Flieg’s score	0.2

**Table 3:** Range analysis of an orthogonal experiment (*n* = 6).

Test Number	Moisture Content (A, %)	Bran Ratio (B, %)	Inoculum Size (C, %)	Blank (D)	FG ^1^ Degradation Rate (%)	pH	NH3-N ^2^/TN ^3^ (%)	NDF ^4^ Degradation Rate (%)	ADF ^5^ Degradation Rate (%)	Flieg’s Score	*F* ^6^
1	1	1	1	1	67.73 ± 4.27	4.81 ± 0.02	10.79 ± 1.84	3.89 ± 0.64	3.06 ± 0.39	64.17 ± 3.97	22.29
2	1	2	2	2	76.58 ± 4.21	4.50 ± 0.05	10.17 ± 0.94	3.12 ± 0.26	3.12 ± 0.73	99.33 ± 1.63	56.53
3	1	3	3	3	79.39 ± 3.78	4.27 ± 0.01	7.99 ± 0.74	3.79 ± 0.53	3.13 ± 0.55	99.17 ± 0.41	75.62
4	2	1	2	3	76.87 ± 2.95	4.83 ± 0.02	12.82 ± 1.26	3.48 ± 0.78	2.86 ± 0.31	66.00 ± 2.00	28.57
5	2	2	3	1	73.85 ± 2.83	4.51 ± 0.02	11.8 ± 1.56	3.28 ± 0.10	2.99 ± 0.72	68.33 ± 2.58	30.42
6	2	3	1	2	76.62 ± 4.46	4.44 ± 0.01	9.88 ± 0.74	2.77 ± 0.39	2.29 ± 0.77	100.00 ± 0.00	48.53
7	3	1	3	2	73.16 ± 5.89	4.92 ± 0.04	10.43 ± 1.18	3.44 ± 0.71	2.94 ± 0.67	68.83 ± 7.31	26.75
8	3	2	1	3	72.24 ± 3.34	4.75 ± 0.01	10.75 ± 0.34	3.98 ± 0.50	2.24 ± 0.43	69.50 ± 6.02	25.81
9	3	3	2	1	72.69 ± 3.72	4.45 ± 0.03	8.93 ± 0.68	5.20 ± 0.32	4.57 ± 0.50	99.83 ± 0.41	81.94
K1	154.44	77.61	96.63	134.65							
K2	107.52	112.76	167.04	131.81							
K3	134.50	206.09	132.79	130.00							
k1	51.48	25.87	32.21	44.88	Order of importance: B > C > A > D						
k2	35.84	37.59	55.68	43.94							
k3	44.830	68.70	44.26	43.33	Best combination: B3C2A1						
R	15.64	42.83	23.47	1.55							

Note: ^1^ FG: Free gossypol; ^2^ NH3-N: ammonia nitrogen; ^3^ TN: total nitrogen; ^4^ NDF: neutral detergent fiber; ^5^ ADF: acid detergent fiber. ^6^ *F*: composite index; it was calculated using the standardized values and weighting coefficients of six fermentation-related indicators, including free gossypol degradation rate, neutral detergent fiber degradation rate, acid detergent fiber degradation rate, ammonia nitrogen/total nitrogen, pH, and Flieg’s score. Higher *F* values indicate better comprehensive fermentation performance.

**Table 4:** Analysis of variance of orthogonal experiment results.

Source	Type III Sum of Squares	df	Mean Square	F	Sig.
Corrected Model	4135.37 ^a^	6	689.23	376.35	<0.01
Intercept	17,464.50	1	17,464.50	9536.44	<0.01
Bran Addition Ratio	826.46	2	413.23	225.65	<0.01
Inoculation Amount	2939.24	2	1469.62	802.48	<0.01
Moisture Content	369.67	2	184.84	100.93	0.01
Error	3.66	2	1.83		
Total	21,603.53	9			

Note: ^a^ indicates the model-fit statistics of the corrected model. R^2^ = 0.999, adjusted R^2^ = 0.996.

**Table 5:** Effect of composite microbial-fermented CSH on sensory scores (*n* = 6).

Item	Group	
	CG ^1^	EG ^2^
Odor	3.33 ± 1.03 ^b^	10.67 ± 0.82 ^a^
Color	1.83 ± 0.41 ^b^	3.00 ± 0.00 ^a^
Texture	1.00 ± 0.00 ^b^	2.00 ± 0.00 ^a^
Total score	6.17 ± 1.17 ^b^	15.67 ± 0.82 ^a^
Grade	Average	Good
Flieg’s score	55.83 ± 28.19 ^b^	98.17 ± 0.41 ^a^
Quality	Average	Excellent

Note: ^1^ CG: Control group; ^2^ EG: Experimental group. Values within the same row followed by different lowercase letters are significantly different (*p* < 0.05), while values followed by the same lowercase letter are not significantly different (*p* > 0.05).

**Table 6:** Effects of composite microbial fermentation on the pH, organic acid and ammonium nitrogen content of CSH (*n* = 6).

Item	Group	
	CG ^1^	EG ^2^
pH	5.01 ± 0.06 ^a^	4.50 ± 0.01 ^b^
Acetic acid (g/kg)	10.27 ± 4.64 ^a^	7.83 ± 0.58 ^b^
Propionic acid (g/kg)	0.92 ± 0.16 ^b^	1.26 ± 0.20 ^a^
Butyric acid (g/kg)	2.47 ± 3.26	-- ^5^
Lactic acid (g/kg)	13.22 ± 5.85 ^b^	33.23 ± 1.74 ^a^
Total acid (g/kg)	26.89 ± 2.75 ^b^	42.32 ± 2.38 ^a^
NH_3_-N ^3^ (g/kg)	1.34 ± 0.15 ^a^	0.76 ± 0.06 ^b^
NH_3_-N/TN ^4^ (%)	9.94 ± 1.26 ^a^	5.73 ± 0.59 ^b^

Note: ^1^ CG: Control group; ^2^ EG: Experimental group; ^3^ NH_3_-N: ammonia nitrogen; ^4^ TN: total nitrogen; ^5^ “--“ indicates not detected. Values within the same row followed by different lowercase letters are significantly different (*p* < 0.05), while values followed by the same lowercase letter are not significantly different (*p* > 0.05).

**Table 7:** Effect of composite microbial fermentation on the nutrient content of CSH (*n* = 6).

Item	Group			*p*-Value
	UG ^1^	CG ^2^	EG ^3^	
DM ^4^ (%)	93.71 ± 0.07	93.82 ± 0.24	93.70 ± 0.54	0.79
CP ^5^ (%)	7.17 ± 0.89 ^b^	8.16 ± 0.19 ^a^	8.37 ± 0.39 ^a^	<0.01
EE ^6^ (%)	1.74 ± 0.14	1.69 ± 0.24	1.83 ± 0.23	0.51
NDF ^7^ (%)	76.10 ± 0.89 ^a^	74.35 ± 3.40 ^a^	70.05 ± 1.96 ^b^	<0.01
ADF ^8^ (%)	44.72 ± 0.91 ^a^	45.17 ± 0.97 ^a^	41.84 ± 0.72 ^b^	<0.01
Ash ^9^ (%)	3.31 ± 0.13 ^c^	3.74 ± 0.21 ^a^	3.53 ± 0.07 ^b^	<0.01
Ca ^10^ (%)	0.18 ± 0.09	0.17 ± 0.04	0.17 ± 0.04	0.97
P ^11^ (%)	0.22 ± 0.02 ^c^	0.39 ± 0.04 ^a^	0.35 ± 0.01 ^b^	<0.01

Note: ^1^ UG: Untreated group; ^2^ CG: Control group; ^3^ EG: Experimental group. ^4^ DM: dry matter; ^5^ CP: crude protein; ^6^ EE: ether extract; ^7^ NDF: neutral detergent fiber; ^8^ ADF: acid detergent fiber; ^9^ Ash: crude ash; ^10^ Ca: calcium; ^11^ P: phosphorus. Dry matter content was determined on an air-dry basis. All nutrient components are expressed on a dry matter basis. Values within the same row followed by different lowercase letters are significantly different (*p* < 0.05), while values followed by the same lowercase letter are not significantly different (*p* > 0.05).

**Table 8:** Effect of composite microbial fermentation of CSH on the levels of FG, aflatoxins and ochratoxins (*n* = 6).

Item	Group			*p*-Value
	UG ^1^	CG ^2^	EG ^3^	
Free gossypol (mg/kg)	166.17 ± 11.56 ^a^	56.94 ± 18.57 ^b^	33.58 ± 6.27 ^c^	<0.01
Aflatoxin B1 (μg/kg)	2.26 ± 0.34 ^b^	3.71 ± 1.32 ^a^	1.48 ± 0.29 ^b^	<0.01
Ochratoxin A (μg/kg)	<10	<10	<10	-

Note: ^1^ UG: Untreated group; ^2^ CG: Control group; ^3^ EG: Experimental group. Values within the same row followed by different lowercase letters are significantly different (*p* < 0.05), while values followed by the same lowercase letter are not significantly different (*p* > 0.05).

## Data Availability

The original contributions presented in this study are included in the article/Supplementary Materials. Further inquiries can be directed to the corresponding author.

## Supplementary Materials

The following supporting information can be downloaded at https://www.mdpi.com/article/10.3390/microorganisms14071456/s1, Figure S1: Representative scanning electron micrographs of cottonseed hulls from the untreated group. Figure S2: Representative scanning electron micrographs of cottonseed hulls from the untreated group2. Figure S3: Representative scanning electron micrographs of cottonseed hulls from the control group. Figure S4: Representative scanning electron micrographs of cottonseed hulls from the control group. Figure S5: Representative scanning electron micrographs of cottonseed hulls from the experimental group. Figure S6: Representative scanning electron micrographs of cottonseed hulls from the experimental group. Table S1: Feature intensity matrix of untargeted metabolomics data in positive ion mode (QC samples)-EG.VS.CG. Table S2: Feature intensity matrix of untargeted metabolomics data in negative ion mode (QC samples)-EG.VS.CG.

## Abbreviations

The following abbreviations are used in this manuscript:

CSH	Cottonseed hulls
UG	Untreated group
CG	Control group
EG	Experimental group
AN	*Aspergillus niger*
AO	*Aspergillus oryzae*
LP	*Lactobacillus plantarum*
FG	Free gossypol
NDF	Neutral detergent fiber
ADF	Acid detergent fiber
NH_3_-N	Ammonia nitrogen
TN	total nitrogen
CP	Crude protein
EE	Ether extract
DEMs	Differential metabolites
UHPLC-MS/MS	Ultra-high-performance liquid chromatography–tandem mass spectrometry
LC-MS	Liquid chromatography–mass spectrometry
FC	Fold change
KEGG	Kyoto Encyclopedia of Genes and Genomes

## References

[1] International Cotton Advisory Committee (ICAC) World Cotton Market Outlook. Presented at WTO Cotton Day, Geneva, Switzerland, 19 November 2025. https://www.wto.org/library/events/event_resources/cott_1911202510/agenda_item_3ia_icac_presentation.pdf.

[2] Elshareef H., Yu Y., Fu Y., Ren S., Tursunov O., Li Y., Dong R., Zhou Y. (2025). Bio-energy potential of cotton stalks via thermal technologies: A review. J. Cotton Res..

[3] Xiong B.H., Luo Q.Y., Zhao F. (2022). Chinese Feed Database Continued. China Feed..

[4] Garleb K.A., Bourquin L.D., Hsu J.T., Wagner G.W., Schmidt S.J., Fahey G.C. (1991). Isolation and chemical analyses of nonfermented fiber fractions of oat hulls and cottonseed hulls. J. Anim. Sci..

[5] Zanine A.M., Castro W.J.R., Ferreira D.J., Souza A.L., Ribeiro M.D., Parente H.N., Parente M.O.M., Santos E.M., Oliveira J.S., Lima A.G.V.O. (2023). Effects of cottonseed hull on intake, digestibility, nitrogen balance, blood metabolites and ingestive behaviour of rams. Sci. Rep..

[6] Mehari T.G., Jiang M., Gu D., Qian J., Tang J., Wubshet A.K., Wang B. (2025). Histopathological examination and transcriptomic profiling reveal gossypol toxicity-responsive genes related to fertility in mice. Front. Pharmacol..

[7] Li J., Gao T., Hao Z., Guo X., Zhu B. (2022). Anaerobic solid-state fermentation with *Bacillus subtilis* for digesting free gossypol and improving nutritional quality in cottonseed meal. Front. Nutr..

[8] Liu B., Liu H., Liu D., Zhou M., Jiang Q., Ma X., Wang J., Tan B., Zhang C. (2024). Free gossypol removal and nutritional value enhancement of cottonseed meal via solid-state fermentation with *Rhodotorula mucilaginosa* TG529. Agriculture.

[9] Liu N., Wang Y., An X., Qi J., Jia Y. (2025). Effects of microbial fermentation on nutrients and flavor substances of cottonseed kernel and functional properties of derived peptides. Chem. Biol. Technol. Agric..

[10] Dong D., Yan Y., Yang F., Yao H., Li Y., Huang X., Aihemaiti M., Zhan F., Hou M., Cui W. (2025). Integrated microbiota and metabolomics analysis of *Candida utilis* CU-3 solid-state fermentation effects on cottonseed hull-based feed. Microorganisms.

[11] Yang X., Sun J.Y., Guo J.L., Weng X.Y. (2012). Identification and proteomic analysis of a novel gossypol-degrading fungal strain. J. Sci. Food Agric..

[12] Li K., Xu Y., Guo K., Cui W., Li Y., Hou M. (2025). Improving the nutritional value and safety of cotton stalk feed via response surface methodology and co-fermentation techniques. Fermentation.

[13] Machida M., Asai K., Sano M., Tanaka T., Kumagai T., Terai G., Kusumoto K.-I., Arima T., Akita O., Kashiwagi Y. (2005). Genome sequencing and analysis of *Aspergillus oryzae*. Nature.

[14] Mageshwaran V., Parvez N. (2016). Gossypol detoxification and lysine enrichment in cottonseed cake by solid state fermentation. J. Pure Appl. Microbiol..

[15] Chen T.M., Li X.M., Zhang G.Y., Zhao H.S., Lu J., Cai G.L. (2024). Effect of *Aspergillus oryzae* fermentation on the feeding quality of cottonseed shells. China Oils Fats.

[16] Dong D., Yao H., Aihemaiti M., Ainizirehong G., Li Y., Yan Y., Huang X., Hou M., Cui W. (2025). Enhanced nutritional composition of steam-exploded cotton stalk through microbial-enzyme synergism solid-state fermentation. Fermentation.

[17] Mageshwaran V., Shaikh A., Kathe A.A. (2017). Optimization of process parameters for gossypol detoxification in chemical disinfected cottonseed cake by mixed fungal culture during solid state fermentation. J. Sci. Ind. Res..

[18] Yusuf H.A., Piao M., Ma T., Huo R., Tu Y. (2021). Enhancing the quality of total mixed ration containing cottonseed or rapeseed meal by optimization of fermentation conditions. Fermentation.

[19] He D., Cui C. (2025). Fermentation of organic wastes for feed protein production: Focus on agricultural residues and industrial by-products tied to agriculture. Fermentation.

[20] Han J., Kamber M., Pei J. (2012). Data Mining: Concepts and Techniques.

[21] Rhine E.D., Mulvaney R.L., Pratt E.J., Sims G.K. (1998). Improving the Berthelot reaction for determining ammonium in soil extracts and water. Soil Sci. Soc. Am. J..

[22] Guo X., Ding W., Yu Z. (2008). Evaluation system for the fermentation quality of silage and recent advances. Chin. J. Grassl. Sci..

[23] Thermo Fisher Scientific (2014). Improved Analysis of Monocarboxylic Acids Using a Thermo Scientific Acclaim Mixed-Mode WAX-1 Column. https://appslab.thermofisher.com/App/772/improved-analysis-monocarboxylic-acids-using-a-thermo-scientific-acclaim-mixedmode-wax1-column.

[24] Hu R., Wu D., Liang X., Wang Z., Zou H., Wu F., Li H., Jiang Y., Peng Q., Xiao J. (2025). Solid state fermentation improves the utilization value of cotton stalk. Ind. Crops Prod..

[25] Latimer G.W., AOAC International (2023). Official Methods of Analysis of AOAC INTERNATIONAL.

[26] Van Soest P.J., Robertson J.B., Lewis B.A. (1991). Methods for dietary fiber, neutral detergent fiber, and nonstarch polysaccharides in relation to animal nutrition. J. Dairy Sci..

[27] Hambleton L.G. (1977). Semiautomated method for simultaneous determination of phosphorus, calcium, and crude protein in animal feeds. J. Assoc. Off. Anal. Chem..

[28] Rahma E.H., Rao M.N. (1984). Gossypol removal and functional properties of protein produced by extraction of glanded cottonseed with different solvents. J. Food Sci..

[29] Want E.J., Masson P., Michopoulos F., Wilson I.D., Theodoridis G., Plumb R.S., Shockcor J., Loftus N., Holmes E., Nicholson J.K. (2013). Global metabolic profiling of animal and human tissues via UPLC-MS. Nat. Protoc..

[30] Smith C.A., Want E.J., O’Maille G., Abagyan R., Siuzdak G. (2006). XCMS: Processing mass spectrometry data for metabolite profiling using nonlinear peak alignment, matching, and identification. Anal. Chem..

[31] Do K.T., Wahl S., Raffler J., Molnos S., Laimighofer M., Adamski J., Suhre K., Strauch K., Peters A., Gieger C. (2018). Characterization of missing values in untargeted MS-based metabolomics data and evaluation of missing data handling strategies. Metabolomics.

[32] Kanehisa M., Furumichi M., Sato Y., Kawashima M., Ishiguro-Watanabe M. (2023). KEGG for taxonomy-based analysis of pathways and genomes. Nucleic Acids Res..

[33] Wishart D.S., Guo A.C., Oler E., Wang F., Anjum A., Peters H., Dizon R., Sayeeda Z., Tian S., Lee B.L. (2022). HMDB 5.0: The Human Metabolome Database for 2022. Nucleic Acids Res..

[34] Conroy M.J., Andrews R.M., Andrews S., Cockayne L., A Dennis E., Fahy E., Gaud C., Griffiths W.J., Jukes G., Kolchin M. (2024). LIPID MAPS: Update to databases and tools for the lipidomics community. Nucleic Acids Res..

[35] Wen B., Mei Z., Zeng C., Liu S. (2017). metaX: A flexible and comprehensive software for processing metabolomics data. BMC Bioinform..

[36] Jiang F., Cheng H., Liu D., Wei C., An W., Wang Y., Sun H., Song E. (2020). Treatment of whole-plant corn silage with lactic acid bacteria and organic acid enhances quality by elevating acid content, reducing pH, and inhibiting undesirable microorganisms. Front. Microbiol..

[37] Chen D., Zhou Y., Yang D., Zhou W., Chen X., Zhang Q. (2023). Exploring *Lactobacillus plantarum* on fermentation quality, gas emissions, and in vitro digestibility of different varieties of litchi leaves silage. Fermentation.

[38] Hu H.L., van den Brink J., Gruben B.S., Wösten H.A.B., Gu J.D., de Vries R.P. (2011). Improved enzyme production by co-cultivation of *Aspergillus niger* and *Aspergillus oryzae* and with other fungi. Int. Biodeterior. Biodegrad..

[39] Song P., Cheng L., Tian K., Zhang M., Mchunu N.P., Niu D., Singh S., Prior B., Wang Z.X. (2020). Biochemical characterization of two new *Aspergillus niger* aspartic proteases. 3 Biotech.

[40] Wei M., Chen P., Zheng P., Tao X., Yu X., Wu D. (2023). Purification and characterization of aspartic protease from *Aspergillus niger* and its efficient hydrolysis applications in soy protein degradation. Microb. Cell Fact..

[41] Feng X., Ng K., Ajlouni S., Zhang P., Fang Z. (2023). Effect of solid-state fermentation on plant-sourced proteins: A review. Food Rev. Int..

[42] Altop A., Güngör E., Erener G. (2019). Improvement of nutritional quality of some oilseed meals through solid-state fermentation using *Aspergillus niger*. Turk. J. Agric. Food Sci. Technol..

[43] Yao Y., Li H., Li J., Zhu B., Gao T. (2021). Anaerobic solid-state fermentation of soybean meal with Bacillus sp. to improve nutritional quality. Front. Nutr..

[44] Wang N., Wang Y., Lin Y., Xu G., Ni K., Yang F. (2023). Effect of lactic acid bacteria and wheat bran on the fermentation quality and bacterial community of Broussonetia papyrifera silage. Chem. Biol. Technol. Agric..

[45] Li X., Pang Y., Zhang R. (2001). Compositional changes of cottonseed hull substrate during *P. ostreatus* growth and the effects on the feeding value of the spent substrate. Bioresour. Technol..

[46] Shi C., He J., Yu J., Yu B., Mao X., Zheng P., Chen D. (2016). Physicochemical properties analysis and secretome of *Aspergillus niger* in fermented rapeseed meal. PLoS ONE.

[47] Jiang Y., Du X., Xu Q., Yin C., Zhang H., Liu Y., Liu X., Yan H. (2023). Biodegradation of gossypol by *Aspergillus* terreus-YJ01. Microorganisms.

[48] Lim S.J., Lee K.J. (2011). A microbial fermentation of soybean and cottonseed meal increases antioxidant activity and gossypol detoxification in diets for Nile tilapia, Oreochromis niloticus. J. World Aquac. Soc..

[49] Mageshwaran V., Sharma V., Chinnkar M., Parvez N., Krishnan V. (2018). Biodegradation of gossypol by mixed fungal cultures in minimal medium. Appl. Biochem. Microbiol..

[50] Jazi V., Boldaji F., Dastar B., Hashemi S.R., Ashayerizadeh A. (2017). Effects of fermented cottonseed meal on the growth performance, gastrointestinal microflora population and small intestinal morphology in broiler chickens. Br. Poult. Sci..

[51] Kumar M., Tomar M., Punia S., Grasso S., Arrutia F., Choudhary J., Singh S., Verma P., Mahapatra A., Patil S. (2021). Cottonseed: A sustainable contributor to global protein requirements. Trends Food Sci. Technol..

[52] Wang L., Huang W., Shen Y., Zhao Y., Wu D., Yin H., Wang J. (2022). Enhancing the degradation of aflatoxin B1 by co-cultivation of two fungi strains with the improved production of detoxifying enzymes. Food Chem..

[53] Wang Y., Jiang L., Zhang Y., Ran R., Meng X., Liu S. (2023). Research advances in the degradation of aflatoxin by lactic acid bacteria. J. Venom. Anim. Toxins Incl. Trop. Dis..

[54] Cater C.M., Lyman C.M. (1969). Reaction of gossypol with amino acids and other amino compounds. J. Am. Oil Chem. Soc..

[55] Ahlberg S.H., Joutsjoki V., Korhonen H.J. (2015). Potential of lactic acid bacteria in aflatoxin risk mitigation. Int. J. Food Microbiol..

[56] De Oliveira D.M., Salvador V.H., Mota T.R., Finger-Teixeira A., de Almeida R.F., Paixão D.A.A., De Souza A.P., Buckeridge M.S., Marchiosi R., Ferrarese-Filho O. (2017). Feruloyl esterase from Aspergillus clavatus improves xylan hydrolysis of sugarcane bagasse. AIMS Bioeng..

[57] Shen P., Zhang Z., He Y., Gu C., Zhu K., Li S., Cao Y. (2018). Magnolol treatment attenuates dextran sulphate sodium-induced murine experimental colitis by regulating inflammation and mucosal damage. Life Sci..

[58] Niu J.L., Wei L.Q., Luo Y.Q., Yang W.T., Lu Q.C., Zheng X.X., Niu Y.J., Sheng W., Cheng H., Zhang W.J. (2021). Fermented cottonseed meal improves production performance and reduces fat deposition in broiler chickens. Anim. Biosci..

[59] Qamar H., He R., Li Y., Song M., Deng D., Cui Y., Yu M., Ma X. (2024). Metabolome and metagenome integration unveiled synthesis pathways of novel antioxidant peptides in fermented lignocellulosic biomass of palm kernel meal. Antioxidants.

