# Supplementary figures and images for: Composite Microbial Solid-State Fermentation Enhances the Fermentation Quality, Nutritional Value, and Safety of Cottonseed Hulls: Insights Based on Physicochemical Detection and Untargeted Metabolomics
Source: Microorganisms. 2026 Jul 2;14(7):1456. doi: 10.3390/microorganisms14071456 (PMC13413447; doi:10.3390/microorganisms14071456)

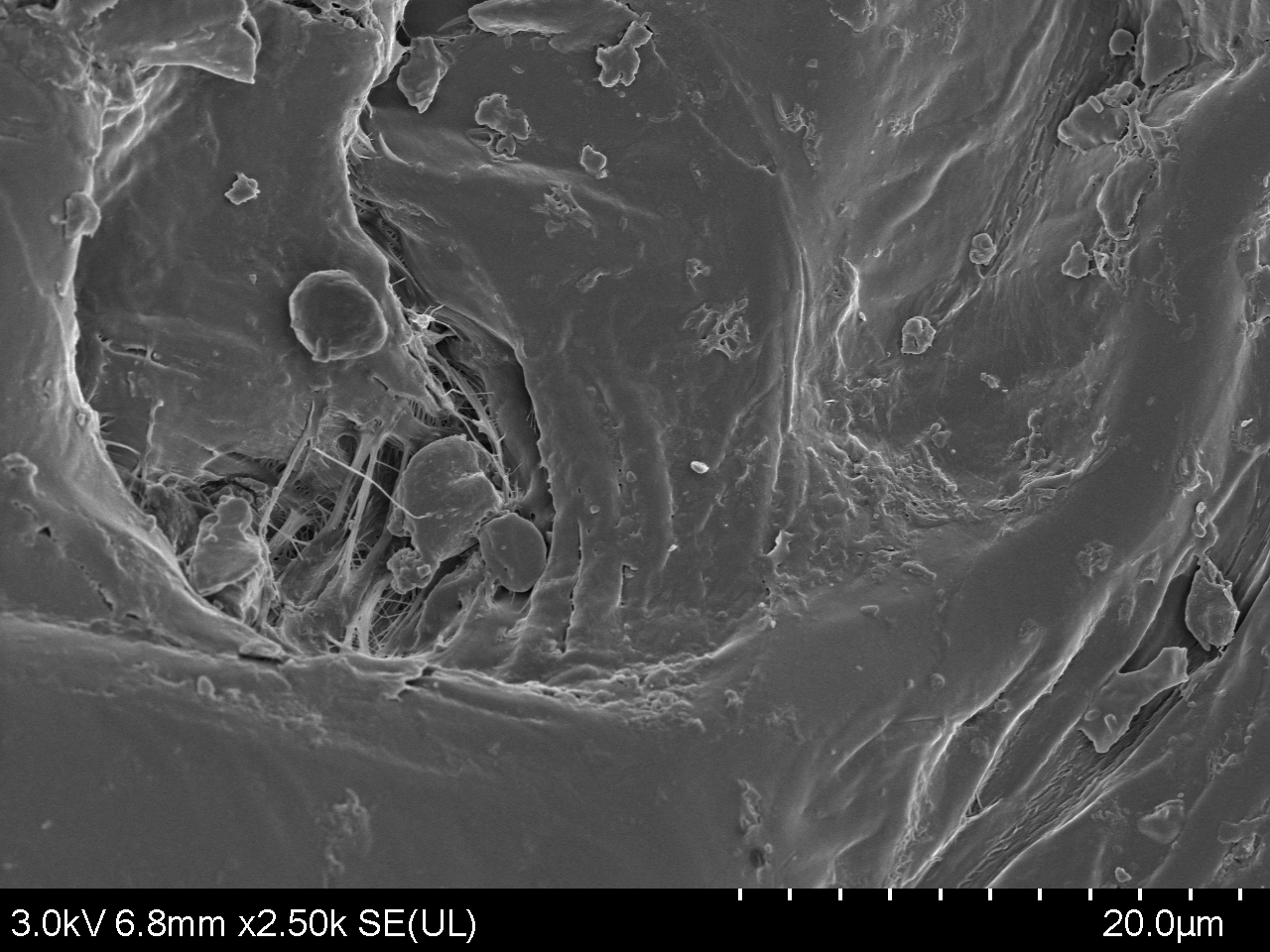

Supplement: Supplementary file 1 [file microorganisms-14-01456-s001.zip › Figure S1. Representative scanning electron micrographs of cottonseed hulls from the untreated group.tif]

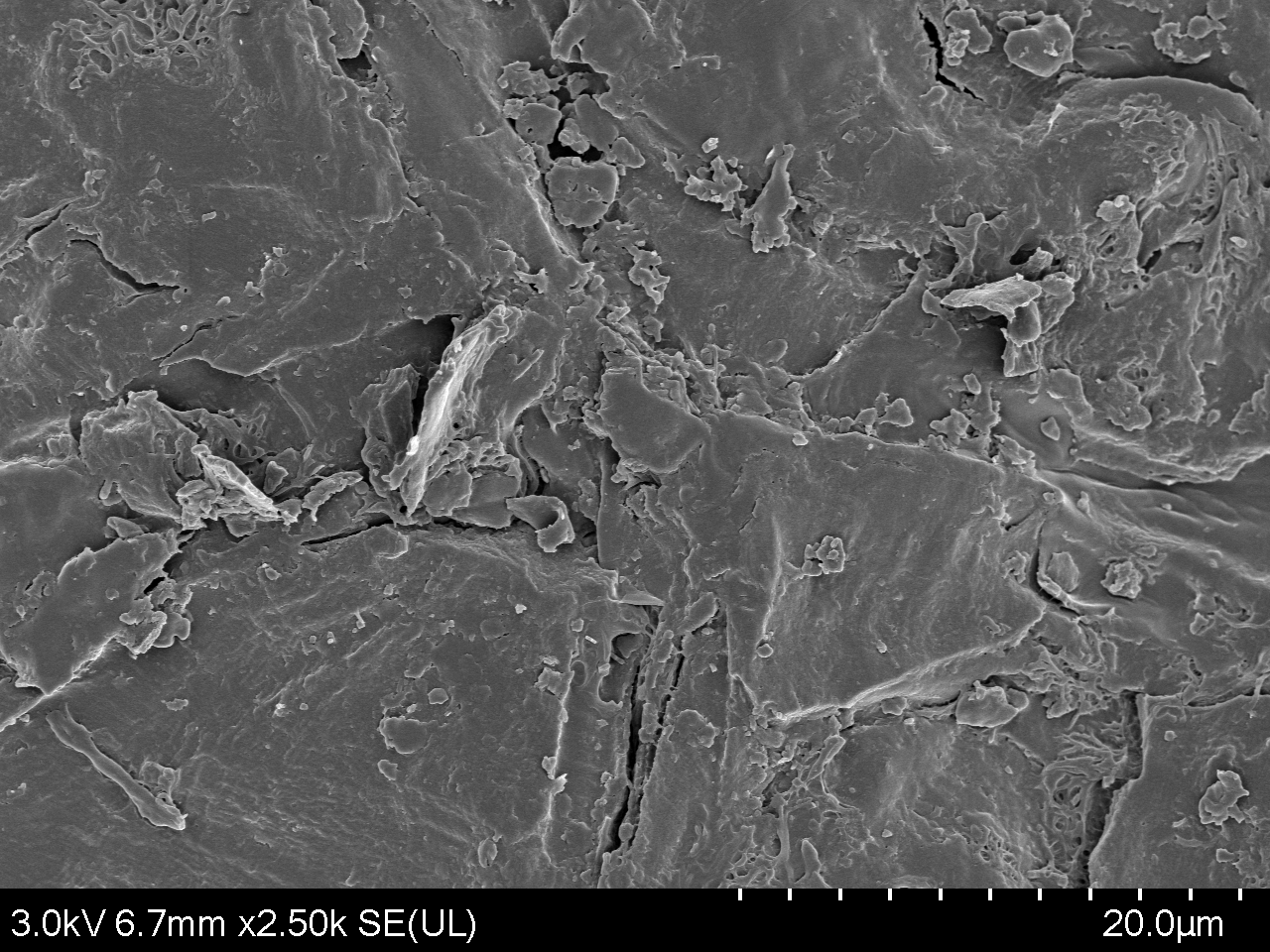

Supplement: Supplementary file 1 [file microorganisms-14-01456-s001.zip › Figure S2. Representative scanning electron micrographs of cottonseed hulls from the untreated group2.tif]

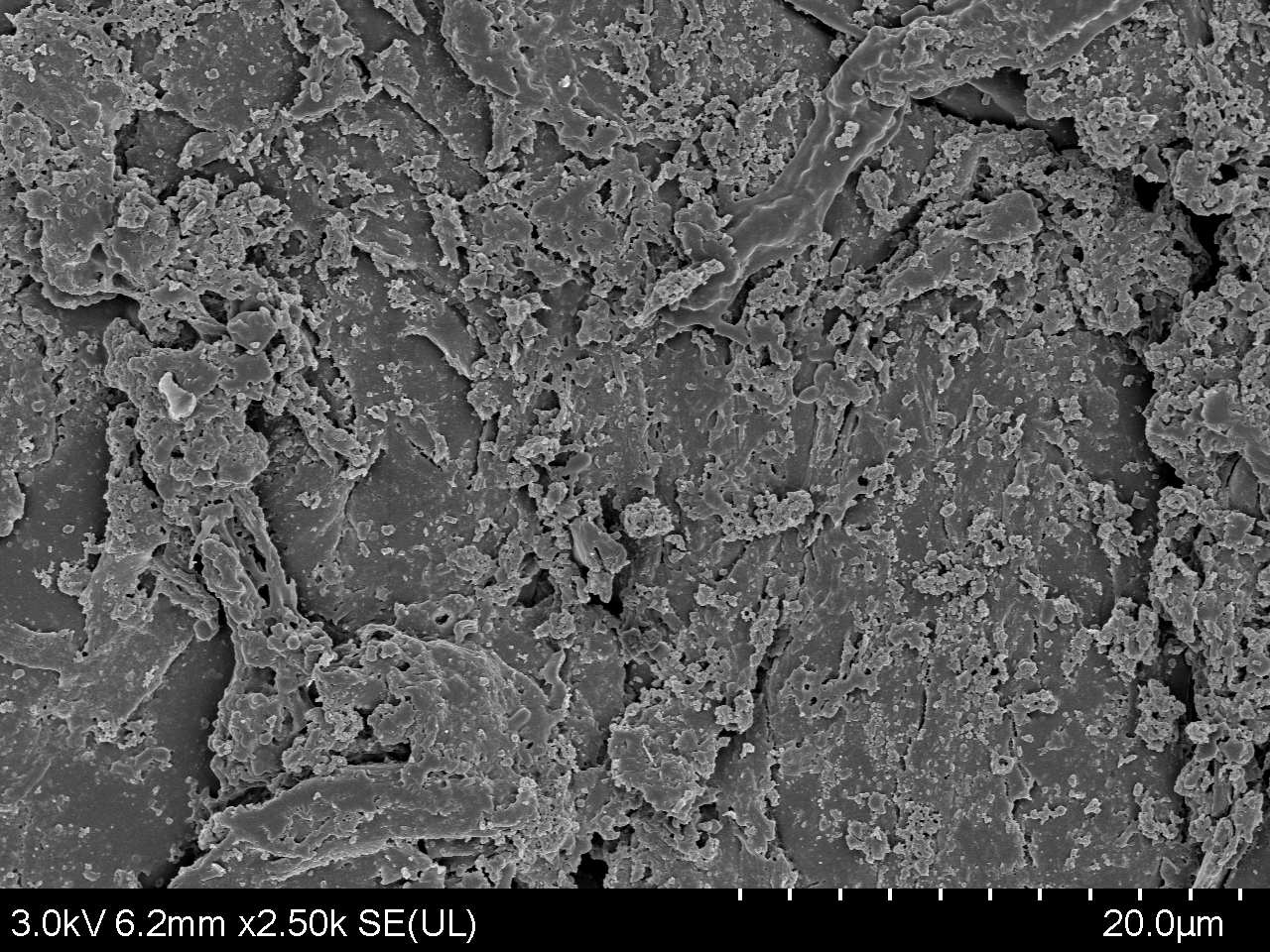

Supplement: Supplementary file 1 [file microorganisms-14-01456-s001.zip › Figure S3. Representative scanning electron micrographs of cottonseed hulls from the control group..tif]

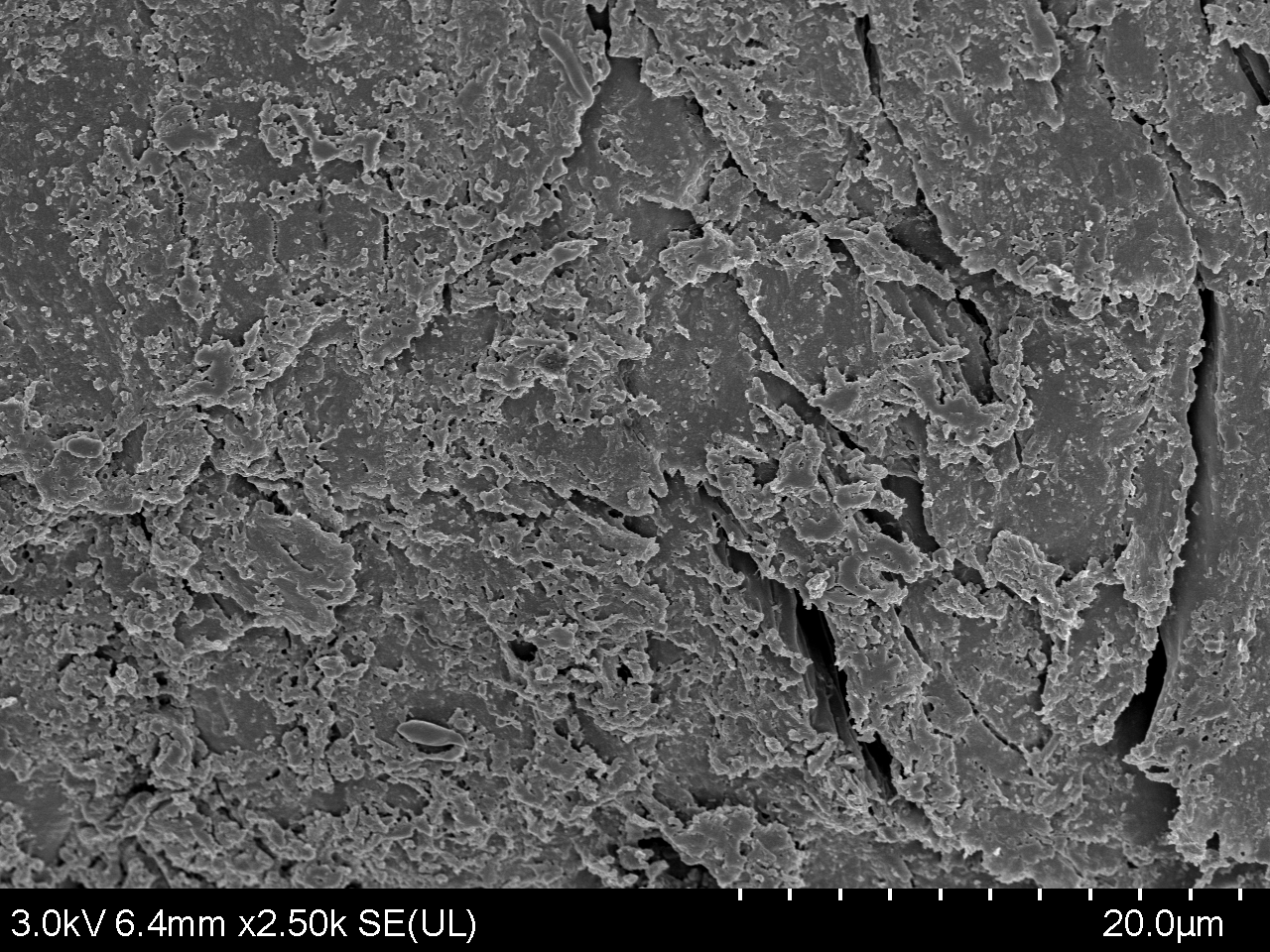

Supplement: Supplementary file 1 [file microorganisms-14-01456-s001.zip › Figure S4 .Representative scanning electron micrographs of cottonseed hulls from the control group..tif]

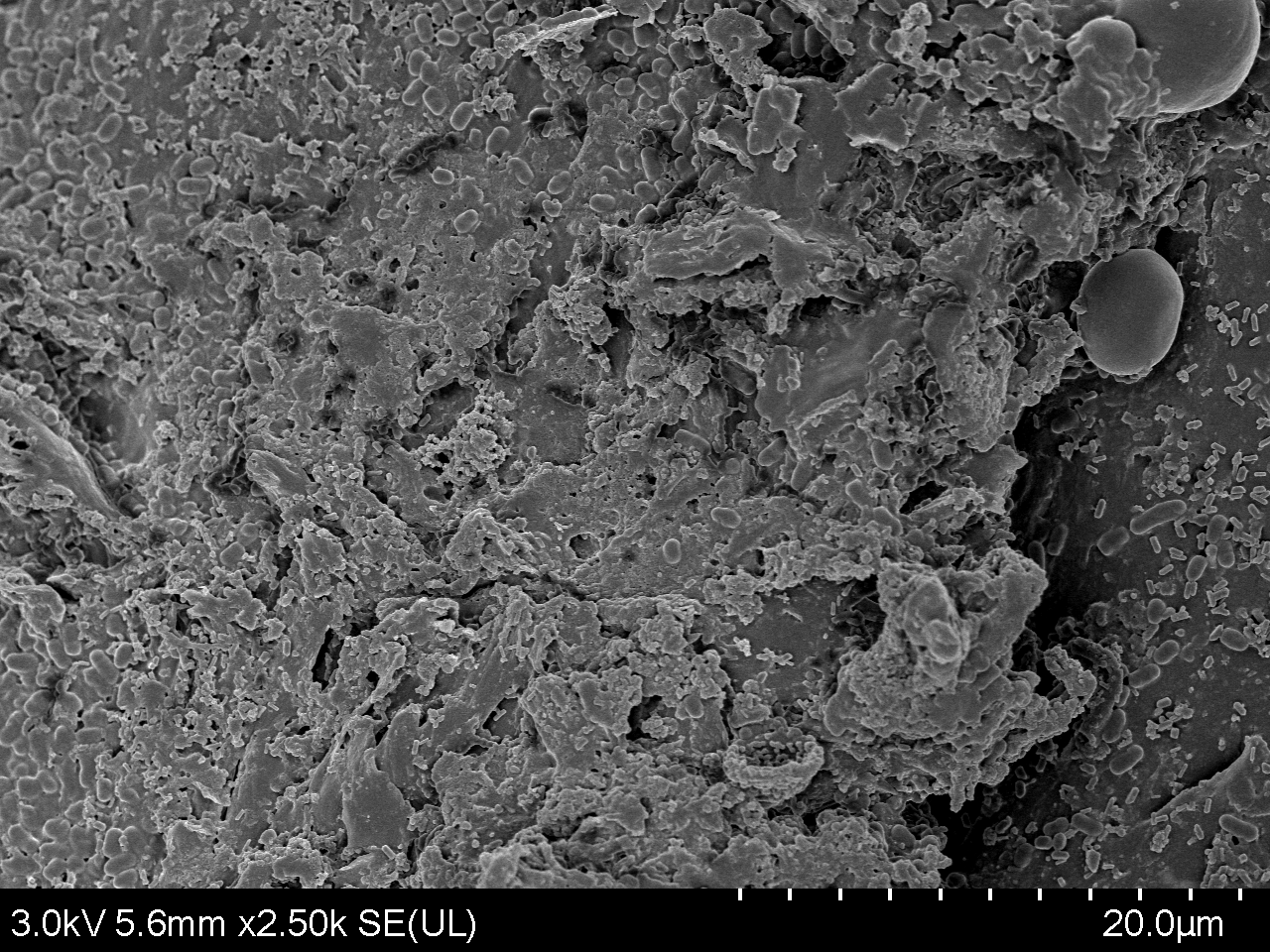

Supplement: Supplementary file 1 [file microorganisms-14-01456-s001.zip › Figure S5. Representative scanning electron micrographs of cottonseed hulls from the experimental group.tif]

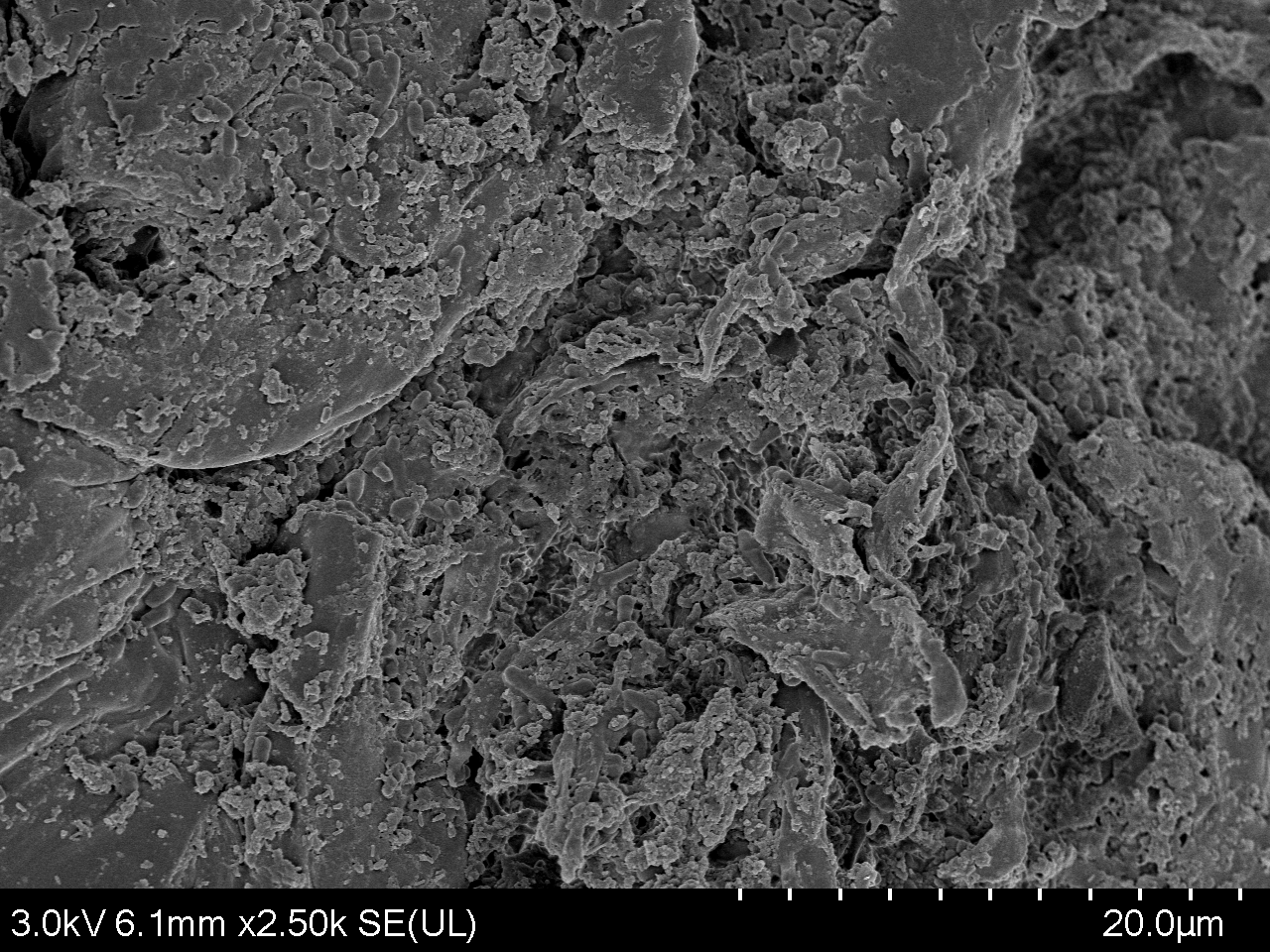

Supplement: Supplementary file 1 [file microorganisms-14-01456-s001.zip › Figure S6. Representative scanning electron micrographs of cottonseed hulls from the experimental group.tif]
